# Development of the gut microbiota in the first 14 years of life and its relations to internalizing and externalizing difficulties and social anxiety during puberty

**DOI:** 10.1007/s00787-023-02205-9

**Published:** 2023-04-18

**Authors:** Yangwenshan Ou, Clara Belzer, Hauke Smidt, Carolina de Weerth

**Affiliations:** 1 Laboratory of Microbiology, Wageningen University and Research, P.O. Box 8033, 6700 EH Wageningen, The Netherlands; 2 Donders Institute for Brain, Cognition and Behaviour, Department of Cognitive Neuroscience, Radboud University Medical Center, P.O. Box 9010, 6500 GL Nijmegen, The Netherlands

**Keywords:** Gut microbiota development, Puberty, Externalizing behavior, Social anxiety, *Prevotella* 9, *Faecalibacterium*

## Abstract

Relations between the gut microbiota and host mental health have been suggested by a growing number of case–control and cross-sectional studies, while supporting evidence is limited in large community samples followed during an extended period. Therefore, the current preregistered study (https://osf.io/8ymav, September 7, 2022) described child gut microbiota development in the first 14 years of life and explored its relations to internalizing and externalizing difficulties and social anxiety in puberty, a period of high relevance for the development of mental health problems. Fecal microbiota composition was analysed by 16S ribosomal RNA gene amplicon sequencing in a total of 1003 samples from 193 children. Through a clustering method, four distinct microbial clusters were newly identified in puberty. Most children within three of these clusters remained in the same clusters from the age of 12 to 14 years, suggesting stability in microbial development and transition during this period. These three clusters were compositionally similar to enterotypes (i.e., a robust classification of the gut microbiota based on its composition across different populations) enriched in *Bacteroides*, *Prevotella*, and *Ruminococcus*, respectively. Two *Prevotella* 9-predominated clusters, including one reported by us earlier in middle childhood and the other one in puberty, were associated with more externalizing behavior at age 14. One *Faecalibacterium*-depleted pubertal cluster was related to more social anxiety at age 14. This finding was confirmed by a negative cross-sectional relation between *Faecalibacterium* and social anxiety in the 14-year-olds. The findings of this study continue to map gut microbiota development in a relatively large community sample followed from birth onwards, importantly extending our knowledge to puberty. Results indicate that *Prevotella* 9 and *Faecalibacterium* may be relevant microbial taxa in relation to externalizing behavior and social anxiety, respectively. These correlational findings need validations from other similar cohort studies, as well as well-designed mechanistic pre-clinical investigations before inferring cause and effect.

**Supplementary Information:**

The online version contains supplementary material available at 10.1007/s00787-023-02205-9.

## Introduction

The gut microbiota plays a critical role in many fundamental aspects of health, and its normal development at early ages is required to maintain host fitness during childhood and later in life [1, 2]. Longitudinal studies have uncovered that the gut microbiota develops in a relatively dynamic pattern in infancy and toddlerhood [3–5]. Importantly, this ecological succession may not come to an end within the first 3 years of life as previously believed [6, 7], but changes continue at least until middle childhood, likely as a result of external influential factors [8]. However, to date, it is unclear if microbial community succession processes continue in puberty, a period with large physical, hormonal, emotional, and social changes.

The gut microbiota is tightly involved in mental health and disorders [9, 10]. Especially early in life, the rapidly developing gut microbiota may influence current and subsequent brain and behavior development along the microbiota–gut–brain axis (MGBA), by activating immune responses, stimulating the vagus nerve, and affecting the host metabolism and endocrine system; and vice versa [9, 10]. It is worth noting that microbiota-behavior relations have been found not only between the gut microbiota in the first 3 years of life and child behavior until age 5 [11–16], but also between early-life gut microbiota and behavior in preadolescents [17]. Children with depleted *Lactobacillales* species at 6 months of age exhibited hyperactivity and impulsivity at age 10 [17]. Such long-lasting associations might be attributed to the impacts of multiple internal (e.g., host biological gender and genetics) and external factors (e.g., antibiotic use and diet) on the gut microbiota in sensitive periods [1, 9, 18].

Puberty is a sensitive developmental window when large physical and mental changes occur. In puberty, children tend to manifest more internalizing and externalizing behavioral difficulties. Internalizing problems influence the internal psychological environment (withdrawal, anxious, and depressive features), while externalizing problems are exhibited in the external environment (impulsive, aggressive, and hyperactive features) [19]. Notably, both types of behavioral difficulties in infancy and middle childhood have been related to gut microbial alpha diversity and relative abundances of individual microbial taxa [8, 13, 16, 20]. Whether similar links exist in puberty remains under-explored until now. In puberty, typically developing children seek to strengthen bonds with their peers and become increasingly independent from their parents [21, 22]. These changes in child behavior increase the risk of developing social anxiety, a complaint that falls under internalizing behavior and plays an important role as a potential antecedent of other internalizing symptoms, such as depression and loneliness [23]. Regarding the MGBA, lower alpha diversity levels and higher *Bacteroides* and *Escherichia*-*Shigella* relative abundances have been reported in patients with generalized anxiety disorder (GAD) [24–26], but information on gut microbial links to social anxiety symptoms in community children in puberty is as yet lacking.

Therefore, our first aim was to describe gut microbiota development from birth to puberty in a longitudinal cohort (*N* = 193 at birth). To this end, pubertal clusters were determined in samples from the ages of 12 and 14 years and combined to the previously determined microbial clusters from infancy (ages 1, 3, and 4 months) and middle childhood (ages 6 and 10 years) [8]. Thereafter, associations between the gut microbiota in the first 14 years of life and internalizing and externalizing difficulties and social anxiety at age 14 were investigated. The associations were analysed in two ways: (1) relations of microbial clusters and phylogenetic diversity over time with child behavioral measures at age 14; (2) cross-sectional relations between individual taxon relative abundances and behavioral measures at age 14.

## Methods

### Study subjects

The study included fecal samples collected at the ages of 1, 3, and 4 months, and 6, 10, 12, and 14 years, from an ongoing longitudinal cohort named BIBO (*N* = 193 originally recruited in pregnancy), with approval from the ethical committee of the Faculty of Social Sciences of Radboud University, Nijmegen, the Netherlands (ECG300107, ECG13012012, SW2017-1303-497 and SW2017-1303-498). The original recruitment criteria and procedures are described elsewhere [27]. The present study was preregistered on the OSF platform (https://osf.io/8ymav).

### Data collection procedures

The original process and criteria of recruitment are described elsewhere [27]. Data collection procedures until age 10 have been described previously [8], and the descriptions of data collection at age 12 can be found through the link (https://osf.io/wu2vt). Fecal microbial composition until age 10 was analyzed previously [8], but not with the aim of relating it to behavioral measures at age 14. Microbial data at age 12 were analyzed in a cross-sectional study (https://osf.io/wu2vt), but not with the aim of describing microbial development and relating it to behavioral measures at age 14. A total of 143 children participated in the round at age 14, of which a number of 125 provided fecal samples. Data of the gut microbiota and behavioral measures at age 14 were not analyzed before preregistration of this study, except for basic descriptive statistics (i.e., distributions, correlations, and internal consistency of behavioral measures). Child characteristics and description as well as their missingness are displayed in Table 1.

**Table 1:** Population characteristics and description at age 14

Variable	Overall, *N* = 143^1^	Boy,* N* = 77^1^	Girl, *N* = 66^1^	*p*^2^	Adjusted *p*^3^
Age in years	14.46 (0.17)	14.46 (0.16)	14.45 (0.19)	0.4	0.7
Thelarche or testicular development	3.81 (0.78)	3.69 (0.80)	3.95 (0.73)	0.044	0.2
Pubarche	3.64 (0.85)	3.35 (0.74)	3.97 (0.86)	< 0.001	< 0.001
zBMI	− 0.09 (1.00)	− 0.18 (1.04)	0.02 (0.95)	0.093	0.2
Sick in the week before the home visit (yes/overall)	7/138 (5.1%)	3/73 (4.1%)	4/65 (6.2%)	0.7	0.8
(Missing)	5	4	1		
Oral antibiotics in the past one year (yes/overall)	2/143 (1.4%)	0/77 (0%)	2/66 (3.0%)	0.2	0.5
Diet quality	86.94 (16.84)	84.06 (17.74)	90.15 (15.29)	0.079	0.2
(Missing)	12	8	4		
Omega-3 fatty acids (yes/overall)	5/138 (3.6%)	2/73 (2.7%)	3/65 (4.6%)	0.7	0.8
(Missing)	5	4	1		
Probiotics (yes/overall)	0/138 (0%)	0/73 (0%)	0/65 (0%)	–	–
(Missing)	5	4	1		
Physical activity	2.34 (0.55)	2.33 (0.57)	2.34 (0.54)	> 0.9	> 0.9
(Missing)	7	5	2		
Drinking alcohol in the past one year (yes/overall)	29/141 (21%)	17/75 (23%)	12/66 (18%)	0.5	0.7
(Missing)	2	2	0		
Smoking cigarettes in the past one year (yes/overall)	4/141 (2.8%)	1/75 (1.3%)	3/66 (4.5%)	0.3	0.6
(Missing)	2	2	0		
Taking drugs in the past one year (yes/overall)	5/141 (3.5%)	2/75 (2.7%)	3/66 (4.5%)	0.7	0.8
(Missing)	2	2	0		
Bristol score^4^	3.12 (0.96)	3.23 (0.91)	2.98 (1.00)	0.058	0.2
(Missing)	23	13	10		
Maternal education level	5.94 (1.33)	5.79 (1.51)	6.12 (1.06)	0.4	0.7
Paternal education level	5.39 (1.83)	5.34 (1.95)	5.45 (1.69)	> 0.9	> 0.9
(Missing)	8	4	4		
Overnight sleep duration in hours	8.24 (1.06)	8.37 (1.12)	8.10 (0.99)	0.086	0.2
(Missing)	6	5	1		
Pets (yes/overall)	96/143 (67%)	49/77 (64%)	47/66 (71%)	0.3	0.6
Internalizing behavior	4.14 (3.03)	3.02 (2.39)	5.44 (3.20)	< 0.001	< 0.001
Min, median, max	0, 4, 16	0, 3, 10	0, 5, 16		
Clinically significant, yes/overall	28/142 (20%)	6/76 (7.9%)	22/66 (33%)		
(Missing)	1	1	0		
Externalizing behavior	6.33 (2.94)	6.16 (3.09)	6.52 (2.76)	0.7	0.8
Min, median, max	0, 6, 15	0, 6, 12	1, 6, 15		
Clinically significant, yes/overall	51/142 (36%)	29/76 (38%)	22/66 (33%)		
(Missing)	1	1	0		
Social anxiety	41.20 (12.62)	38.14 (12.17)	44.63 (12.32)	0.001	0.008
Min, median, max	18, 39, 79	18, 36, 79	18, 43, 71		
Clinically significant, yes/overall	34/138 (25%)	13/73 (18%)	21/65 (32%)		
(Missing)	5	4	1		

^1^Mean (SD); *n*/*N* (%)

^2^Wilcoxon rank sum test; Fisher's exact test; Pearson's Chi-squared test

^3^False discovery rate correction for multiple testing

^4^Bristol stool consistency scale was used as a numeric variable

### Gut microbiota composition

Regarding the fecal samples at age 14, we used the same DNA isolation protocol as used for earlier samples [8]. In brief, 0.01 to 0.13 g of fecal samples were used for microbial DNA extraction through the Maxwell 16 Total RNA system (Promega, Wisconsin, USA). Duplicate amplicons of the V4 region of bacterial and archaeal 16S ribosomal RNA (rRNA) genes were purified and adjusted to 200 ng per sample prior to being sequenced.

The sequence data in puberty (i.e., *N* = 139 and 125 samples available at the ages of 12 and 14, respectively) were included and processed using the NG-Tax 2.0 pipeline to identify amplicon sequence variants (ASVs) [28, 29]. Those ASVs were taxonomically assigned based on the SILVA_138_SSU 16S rRNA gene reference database [30]. A total of 52,054,996 reads were obtained, with a median of 182,740 reads per sample. Taxa observed in puberty were used in microbial cluster identification and behavioral relation investigation as outlined below. Regarding microbial data until age 10 (i.e., *N* = 739 samples at ages of 1, 3, and 4 months, and 6 and 10 years), we directly used the microbial clusters (i.e., three clusters in infancy and four clusters in middle childhood) and phylogenetic diversity presented in our earlier study [8].

### Behavioral measures

Children at age 14 were asked to fill in the Strengths and Difficulties Questionnaire (SDQ) for internalizing and externalizing difficulties [31] and the Social Anxiety Scale for Adolescents (SAS-A) for their social anxiety complaints [32]. The SDQ includes internalizing and externalizing subscales. Each subscale includes ten items, scored on a 3-point scale (0 to 2), leading to a final score ranging from 0 to 20. The SAS-A includes 18 items used for anxiety evaluation and 4 filler items not used for calculating the score. Each SAS-A item is scored on a 5-point scale (1 to 5), leading to a total social anxiety score ranging from 18 to 90. Higher scores on internalizing and externalizing behavior, and social anxiety reflect more difficulties. The cut-offs for clinical behavioral problems in community samples are: internalizing behavior > 7 [33], externalizing behavior > 8 [33], and social anxiety ≥ 50 [34, 35]. These behavioral measures were confirmed to have acceptable internal consistency represented by *ω*_total_ values [36], namely: internalizing = 0.71, externalizing = 0.68, and social anxiety = 0.94, as calculated with the *psych* R package [37]. Internalizing behavior and social anxiety were highly correlated (Spearman’s Rho = 0.72, *p* < 0.001), while externalizing behavior was not correlated to internalizing behavior and social anxiety (Spearman’s Rho = 0.11 and 0.10, respectively).

### Potential covariates

We also measured variables known to be related to the gut microbiota and host behavior, at child age 14: (1) age in years; (2) child gender (boy and girl); (3) tanner stages, including thelarche or testicular development and pubarche (both are self-assessed on a 5-point scale, with score 1 indicating a prepubertal status and score 5 referring to complete sexual maturity); (4) zBMI calculated with the WHO Growth Reference via the *zscore* R package [38]; (5) whether a child was sick in the week before the home visit [27]; (6) whether a child took antibiotics in the past 1 year [27]; (7.1) diet quality, measured by an online self-report questionnaire named *Eetscore* [39], which assesses the adherence to the Dutch dietary guideline. The total score can range from 0 to 160 points, with higher scores representing better adherence to the guideline and hence a generally healthier diet; (7.2) consumption of omega-3 fatty acids; (7.3) consumption of probiotics; (8) physical activity, measured by Physical Activity Questionnaire for Adolescents (PAQ-A) [40]. The final PAQ-A activity summary score ranges from 1 to 5, with score 1 indicating low physical activity and score 5 indicating high physical activity; (9) the use of alcohol, tobacco, and drugs, measured by Brief Screener for Tobacco, Alcohol, and Other Drugs (BSTAD) [41]; (10) stool consistency as measured by the 7-point Bristol stool scale, with type 1 indicating the most lumpy and type 7 referring to the most liquid [42]. Types 3 and 4 (i.e., sausage- or snake-like with either cracks on surface or being smooth and soft) are considered as normal stool types in general populations [43]; (11) maternal and paternal education levels ranging from 1 to 8, with higher scores indicating higher levels of education; (12) overnight sleep duration in hours, measured by the Pittsburg Sleep Quality self-report questionnaire [44]; (13) pets (yes or no).

### Statistical analyses

All analyses were performed in R studio (version 4.1) with the *phyloseq*, *microbiome*, *picante*, *dplyr*, *data.table*, *tidyr*, *moments*, *faraway*, *gtsummary*, *ComplexHeatmap*, *ggpubr*, *microbiomeMarker*, and *MASS* R packages.

#### First aim: gut microbiota development in the first 14 years of life

We used microbial clusters (i.e., conserved compositional patterns of the gut microbiota) to describe gut microbiota development from birth until age 14. Microbial clusters from birth until age 10 were identified through Dirichlet multinomial mixture (DMM) models [45] in our earlier research [8], and therefore we directly included them in the current study. Here, we analyzed microbial data at ages 12 and 14 together by using the same clustering methods. The optimal number of pubertal microbial clusters was determined by the lowest Laplace approximation score.

Development and transition of pubertal microbial clusters were displayed together with the infant and childhood microbial clusters reported previously. ASV-based phylogenetic diversity and genus-level beta diversity (using weighted UniFrac distance) were compared between pubertal microbial clusters. LEfSe (i.e., Linear discriminant analysis Effect Size) was used to identify differentially abundant microbial taxa between pubertal clusters. Multiple comparisons were corrected with the false discovery rate (FDR) method.

Additionally, we assessed if pubertal microbial clusters were associated with the potential covariates aforementioned. To this end, we used redundancy analysis (RDA) to evaluate to what extent the microbial variance at age 14 was explained by potential covariates. Both simple and marginal effects were measured. Simple effects refer to variance explained by one variable without considering any other variables. Marginal effects mean variance explained by one variable after variance explained by other variables was taken out.

#### Second aim: associations between the gut microbiota across the first 14 years of life and behavioral measures at age 14

Generalized linear models (GLMs) were implemented to assess relations of microbial clusters and phylogenetic diversity over time with behavioral measures (i.e., internalizing and externalizing behavior, and social anxiety) at age 14. Notably, the gut microbiota develops rapidly in early life [4, 6], and the microbiota at various ages may indicate different relations to behavioral outcomes [46]. Therefore, we first conducted analyses of microbial data at each age separately (i.e., ages of 1, 3, and 4 months, and 6, 10, 12, and 14 years). Second, we carried out analyses at the different developmental stages (i.e., infancy, childhood, and puberty), as these collapse the individual time points to provide broader windows (e.g., infancy, childhood, and puberty) during which the gut microbiota may impact behavior. Both analyses can provide insight into potential sensitive time points and windows for the gut microbiota to influence development. Additionally, GLMs were also conducted to measure cross-sectional relations between individual taxon relative abundances at the genus level and the behavioral measures at age 14. We also described how much microbial variance at age 14 was explained by behavioral measures at the same age through RDA.

To select the best fitting distributions for behavioral outcomes, we measured their distribution normality and skewness. Internalizing behavior and social anxiety were right-skewed (skewness = 0.97 and 0.60) and non-normally distributed (normality assessed by the Shapiro–Wilk test, *p* < 0.01 for both, indicating non-normal distribution), and therefore negative binomial distribution was used in GLMs [47]. Externalizing behavior was normally distributed (*p* = 0.08 > 0.05) and not skewed (skewness = 0.12), so the normal distribution was used in GLMs.

Two different models were conducted as follows:

1. A crude model of *B*_*i*_ ~ *M*_*j*_ was used to measure the independent relation between behavioral measures and microbial parameters. “*B*_*i*_” represents the matrix of behavioral measures, with “_*i*_” referring to either internalizing or externalizing behavior, or social anxiety. “*M*_*j*_” indicates microbial parameters, with “_*j*_” being either microbial clusters, phylogenetic diversity, or relative abundances of an individual genus-level taxon prevalent in more than 10% of 14-year-old samples.
2. An adjusted model of *B*_*i*_ ~ *M*_*j*_ + *potential covariates* was implemented when its corresponding crude model was found to have an original unadjusted *p* < 0.05. Before conducting adjusted models, we assessed independent relations between the behavioral measures and their potential covariates with GLMs (Table S1). Those with original *p* < 0.05 were used in the adjusted models [48], including: (a) child gender, diet quality, and overnight sleep duration were included for internalizing behavior; (b) overnight sleep duration and alcohol intake were included for externalizing behavior; (c) child gender, diet quality, overnight sleep duration, and paternal education levels were included for social anxiety. The variance inflation factor (VIF) values of *M*_*j*_ and potential covariates in all adjusted models were less than three, indicting no multicollinear issues [49].

Multiple GLM tests were corrected by FDR methods.

#### Significance

The significance was defined as *p* < 0.05 for non-multiple tests or FDR-adjusted *p* < 0.05 for multiple tests, except for RDA of which significances were determined by permutation tests.

## Results

### Population characteristics and descriptives

Approximately half of the children participating in the round of age 14 were boys (Table 1). Compared to boys, girls developed significantly quicker in sexual maturity and had more self-reported internalizing behavior and social anxiety. Furthermore, girls exhibited insignificant but slightly higher zBMI values, better diet quality, lower Bristol scores (the distribution of Bristol stool consistency types in categorical format is displayed in Figure S1), and fewer sleeping hours (unadjusted *p* < 0.10). Regarding microbial variance explained by potential covariates (Table S2; significances were determined by permutation tests without FDR adjustments), overnight sleep duration accounted for 3.05% total variation (simple effect, *p* < 0.01), followed by drinking alcohol (simple effect, *R*^2^% = 1.72% but insignificant with *p* = 0.07). The significance remained for overnight sleep duration after partitioning the variance explained by other variables (marginal effect, *R*^2^% = 2.07% and *p* = 0.03).

### Gut microbiota development in the first 14 years of life

#### Microbial clusters and their transition

**Fig. 1:**
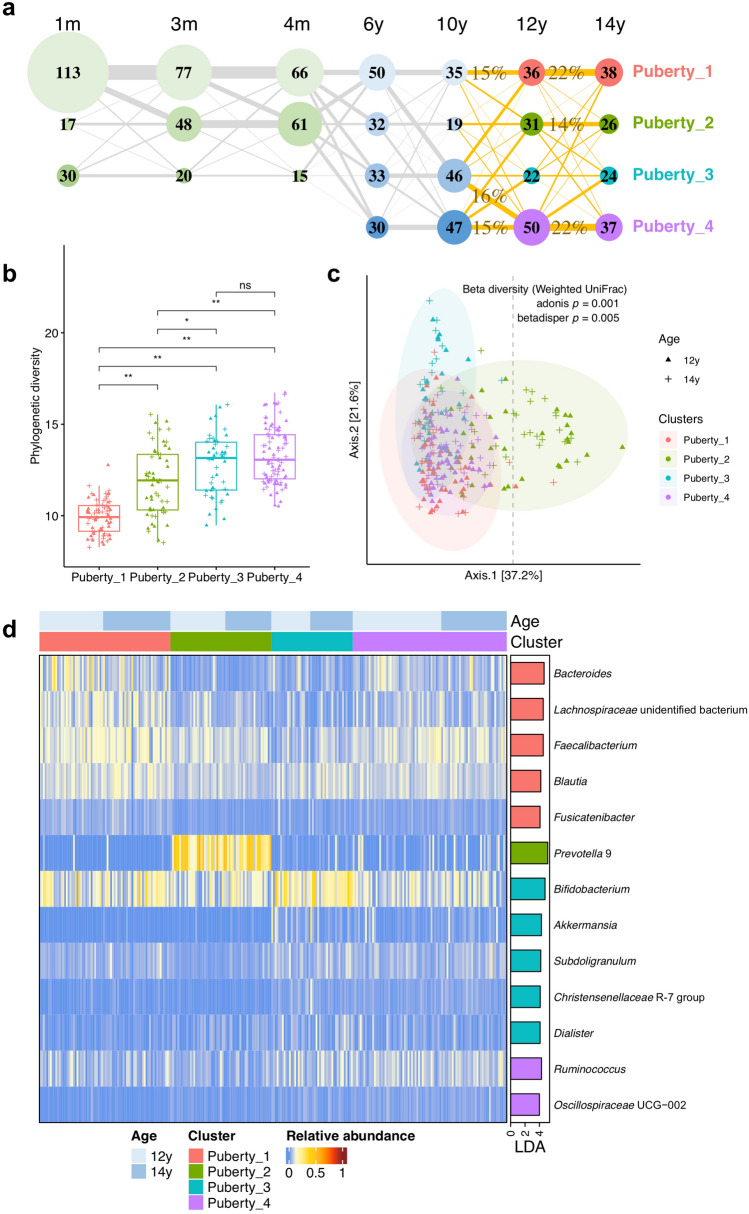
Microbial clusters. **a** Transition between microbial clusters in the first 14 years of life. Microbial clusters were determined by the DMM clustering method according to their compositional characteristics at the genus level. The clusters in infancy (i.e., ages of 1, 3, and 4 months) and middle childhood (i.e., ages of 6 and 10 years) were reported previously [8], and the clusters in puberty (i.e., ages of 12 and 14 years) were determined in the present study. Microbial clusters are presented as nodes, with the size and the number indicating how many samples belong to the corresponding cluster. The four pubertal clusters are colored in pink, grass green, lake blue, and purple, respectively. Transition rates between clusters were calculated based on complete case samples and are shown as sized lines. There are *N* = 130 completed cases between age 10 and age 12, and *N* = 116 completed cases between age 12 and age 14. The lines from ages 10 to 14 are highlighted in orange, accompanied with transition rates (> 10%) in percentages. **b** Phylogenetic diversity of pubertal microbial clusters. Box plots show interquartile ranges and median values. Whiskers indicate 1.5 times the interquartile ranges. Wilcoxon tests were implemented between clusters with the FDR correction (adjusted *p*: ns, not significant; * < 0.05; ** < 0.01). **c** Beta diversity between pubertal microbial clusters. It was calculated by weighted UniFrac distance based on relative abundance data of genus-level microbial taxa. Ellipses represent 95% confidence intervals for pubertal clusters assuming a multivariate normal distribution. **d** Differentially abundant genus-level taxa between microbial clusters in puberty. These taxa were identified through LEfSe (Linear discriminant analysis Effect Size) with FDR-adjusted *p* < 0.05 and LDA (Linear discriminant analysis) effect size > 4. Taxon relative abundances in individuals are shown in the heatmap on the left side. The barplot on the right side represents LDA scores, with colors indicating enriched clusters

Four microbial clusters were identified from *N* = 264 pubertal samples at the ages of 12 and 14 years based on their compositional features (Fig. 1a), determined by the lowest Laplace value in DMM models (Figure S2). No significant differences were observed in potential covariates between these clusters after FDR corrections (Table S3). However, Puberty_2 and Puberty_4 tended to include more boys (67%, 38/57 boys in Puberty_2; 60%, 52/87 boys in Puberty_4), and Puberty_3 consisted of fewer boys (37%, 17/46); Pearson's Chi-squared test *p* = 0.009 and adjusted *p* = 0.2. Besides, children within Puberty_1 likely took more oral antibiotics (8.1%, 6/74 had oral antibiotics in Puberty_1, and less than 5% in the other three clusters); Fisher's exact test *p* = 0.034 and adjusted *p* = 0.2. Furthermore, 38% (10/26) of 14-year-old children within Puberty_2 drank alcohol in the past 1 year, which was more frequent than those belonging to other pubertal clusters at this age (less than 20%); Fisher's exact test* p* = 0.016 and adjusted *p* = 0.2.

At age 12, 26%, 22%, 16%, and 36% (36, 31, 22, and 50/139) of children belonged to microbial clusters Puberty_1, Puberty_2, Puberty_3, and Puberty_4, respectively. At age 14, 30%, 21%, 19%, and 30% (38, 26, 24, and 37/125) of children belonged to these four pubertal clusters, respectively.

In puberty, *N* = 116 children provided both samples at the ages of 12 and 14. Of these children, 22% remained in Puberty_1, another 22% remained in Puberty_4, and 14% remained in Puberty_2 (Fig. 1a). Furthermore, 81% (25/31) of children in Puberty_1 at age 12 stayed in the same cluster at age 14, and 62% (16/26) in Puberty_2, and 59% (26/44) in Puberty_4, suggesting a more stable transition within Puberty_1 compared to Puberty_2 and Puberty_4 during this period (Table S4). In contrast, children within Puberty_3 at age 12 showed a more diverse developing track from ages 12 to 14. Among *N* = 130 completed cases at the ages of ten and 12, 16% and 15% of these children transitioned from childhood microbial clusters 3 and 4 to Puberty_4, while 15% of them developed from childhood cluster 1 to Puberty_1 (Fig. 1a; transition rates are shown in more detail in Table S4).

#### Compositional features of pubertal microbial clusters

Puberty_1 showed the lowest phylogenetic diversity followed by Puberty_2, and Puberty_3 and Puberty_4 exhibiting the highest phylogenetic diversity (Fig. 1b). The ellipses of Puberty_1, Puberty_2, and Puberty_3 partially overlapped with each other, and the ellipse of Puberty_4 was almost completely within the overlapping part of aforementioned three clusters (Fig. 1c). Furthermore, the ellipse of Puberty_2 was larger than the ellipses of the other three clusters, suggesting more interpersonal variations in the gut microbiota of Puberty_2. Besides, we observed different compositional variances (i.e., heterogeneity of dispersion) among pubertal clusters (betadisper *p* = 0.005). Specifically, Puberty_4 differed from Puberty_2 and Puberty_3 (betadisper *p* = 0.001 for both). The adonis function (*p* = 0.001) further showed general dissimilarity in microbial composition between pubertal clusters. Pairwise comparisons between clusters confirmed this result (adonis *p* = 0.001 for all). Additionally, we found 31 samples within Puberty_2 (including *N* = 14 at age 12 and *N* = 17 at age 14) located dispersedly (as shown on the right side of the vertical dashed line), in comparison with other samples in puberty.

Nine, 15, 28, 43, and 105 microbial taxa were found differentially abundant between pubertal clusters at the levels of phylum, class, order, family, and genus, based on LEfSe analysis (effect size > 2 and FDR-adjusted *p* < 0.05), respectively. Particularly, Puberty_1 was enriched in *Bacteroides*, an unidentified genus within *Lachnospiraceae* family, *Faecalibacterium*, *Blautia*, and *Fusicatenibacter*, Puberty_2 was predominated by *Prevotella* 9, Puberty_3 was enriched in *Bifidobacterium*, *Akkermansia*, *Subdoligranulum*, *Christensenellaceae* R-7 group, and *Dialister*, and Puberty_4 was enriched in *Ruminococcus* and *Oscillospiraceae* UCG-002 (Fig. 1d).

### Associations between the gut microbiota across the first 14 years of life and behavioral measures at age 14

#### Relations of microbial clusters and phylogenetic diversity over time with child behavioral measures at age 14

First, independent relations between microbial predictors at each time point or period (i.e., either microbial clusters or phylogenetic diversity in infancy including 1, 3, and 4 months, childhood including 6 and 10 years, or puberty including 12 and 14 years) and behavioral outcomes at age 14 (i.e., internalizing and externalizing behavior, and social anxiety) were determined by crude generalized linear models (GLMs), without accounting for any covariates. Next, we adjusted GLMs with potential covariates for the behavioral outcomes. This was based on covariates that displayed original *p* values lower than 0.05 in crude GLMs (Table 2; See detailed GLM results regarding clusters and phylogenetic diversity in Tables S5 and S6, respectively).

**Table 2:** Main findings of the relations between either microbial clusters or phylogenetic diversity in the first 14 years of life and behavioral outcomes at age 14

Age	Cluster or diversity	Crude model			Adjusted model			
		Estimate (Std. error)	*p*	Adjusted* p*	Estimate (Std. error)	*p*	Adjusted* p*	VIF
Internalizing behavior								
Infancy	Infancy_2	0.2 (0.1)	0.044	0.102	0.2 (0.1)	0.007	0.021	1.02
Infancy	Phylogenetic diversity	< 0.1 (< 0.1)	0.016	0.030	< 0.1 (< 0.1)	0.002	0.004	1.03
Externalizing behavior								
6y	Childhood_2	1.9 (0.7)	0.008	0.023	1.6 (0.7)	0.025	0.060	1.13
12y	Puberty_2	1.9 (0.7)	0.012	0.033	1.7 (0.7)	0.021	0.054	1.06
14y	Puberty_2	1.8 (0.7)	0.012	0.034	1.0 (0.7)	0.190	0.308	1.18
Childhood	Childhood_2	1.7 (0.5)	0.002	0.009	1.4 (0.5)	0.008	0.023	1.07
Puberty	Puberty_2	1.9 (0.5)	< 0.001	0.001	1.3 (0.5)	0.010	0.029	1.08
Social anxiety								
3 m	Infancy_2	0.1 (0.1)	0.033	0.078	0.2 (0.1)	0.003	0.012	1.08
14y	Puberty_3	0.2 (0.1)	0.017	0.044	0.2 (0.1)	0.015	0.040	1.17
Puberty	Puberty_3	0.1 (0.1)	0.048	0.107	0.2 (0.1)	0.004	0.012	1.11

Only clusters or phylogenetic diversity with original *p* < 0.05 in crude GLMs are displayed here. As microbial cluster is a categorical variable, comparisons were implemented between the first cluster and other clusters at the corresponding time point or period. Phylogenetic diversity was used as a numeric variable. In adjusted models, child gender, diet quality, and overnight sleep duration were included for internalizing behavior; overnight sleep duration and alcohol intake were included for externalizing behavior; and child gender, diet quality, overnight sleep duration, and paternal education levels were included for social anxiety. VIF < 3 indicates no multicollinearity in adjusted models

In adjusted GLMs, we observed increased internalizing behavior in cluster Infancy_2 in the period from ages 1 to 4 months (estimate = 0.2 and adjusted *p* = 0.021), but not at separate time points. Similarly, we found increased externalizing behavior in Childhood_2 and Puberty_2 during their corresponding periods in adjusted GLMs (estimates = 1.4 and 1.3, respectively; adjusted *p* = 0.023 and 0.029, respectively). Besides, increased social anxiety was found in Infancy_2 at the age of three months and Puberty_3 at the age of 14 years and in the period of puberty (estimates ≤ 0.2 and adjusted *p* < 0.05 after accounting for covariates). With respect to phylogenetic diversity, the only significant finding was observed in infancy, with a mildly positive relation to social anxiety at age 14 (estimate < 0.1; adjusted *p* = 0.004 in the adjusted GLM), i.e., increased phylogenetic diversity in infancy was related to increased social anxiety difficulties at age 14.

Additionally, we explored differences in behavioral relations between disperse Puberty_2 samples and other samples in puberty based on beta diversity (Table S7). To this end, we performed the same crude and adjusted GLMs described above. Disperse Puberty_2 samples at age 14 showed significantly more internalizing behavior at the same age without accounting for covariates (estimate = 0.4 and adjusted *p* = 0.02), while the difference became marginally insignificant after considering covariates (estimate = 0.3 and adjusted *p* = 0.079). Similarly, after partialling out potential influences of covariates, disperse Puberty_2 samples in the period of puberty did not exhibit increased externalizing behavior (crude GLM: estimate = 1.3 and adjusted *p* = 0.041; adjusted GLM: estimate = 0.6 and adjusted *p* = 0.338).

#### Cross-sectional relations between the gut microbiota and behavioral measures in 14-year-old children

RDAs showed that externalizing behavior was the only behavioral measure that significantly explained microbial variance in the 14-year-olds without considering other variables (simple effect, *R*^2^% = 1.93% and *p* = 0.04; Table S2). However, after partitioning the variance explained by overnight sleep duration and drinking alcohol, externalizing behavior did not remain significant (marginal effect, *R*^2^% = 0.58% and *p* = 0.71). We further measured cross-sectional relations between relative abundances of individual genus-level taxa and the behavioral measures at age 14. Table 3 presents the results of taxa in which the original significance in crude GLMs was* p* < 0.05.

**Table 3:** Main findings of the relations between taxon relative abundances at the genus-level and behavioral measures in children at age 14

Genus	Crude model			Adjusted model				Fold change of estimates (crude/adjusted)	Mean of relative abundance (SD) %	Prevalence %
	Estimate (Std. Error)	*p*	Adjusted *p*	Estimate (Std. error)	*p*	Adjusted *p*	VIF			
Internalizing behavior										
*Agathobacter*	81.1 (34.4)	0.020	0.038	49.3 (32.5)	0.132	0.212	1.02	1.65	0.1 (0.2)	44.8
*Barnesiella*	− 28.4 (12.3)	0.023	0.044	− 20.3 (11.1)	0.071	0.123	1.02	1.40	0.4 (0.5)	70.4
*Collinsella*	− 9.6 (4.7)	0.045	0.083	− 4.1 (4.3)	0.340	0.455	1.07	2.34	0.7 (1.4)	52.0
*Faecalibacterium*	− 3.1 (1.2)	0.012	0.023	− 2.2 (1.2)	0.073	0.126	1.12	1.41	9.3 (5.0)	99.2
*Intestinibacter*	17.5 (8.4)	0.039	0.073	14.6 (7.7)	0.060	0.106	1.01	1.20	0.6 (0.7)	72.8
*Lachnospira*	36.4 (15.0)	0.017	0.033	18.9 (18.4)	0.305	0.421	1.07	1.93	0.3 (0.4)	57.6
*Turicibacter*	31.8 (13.7)	0.022	0.043	26.0 (12.5)	0.039	0.073	1.01	1.22	0.2 (0.4)	32
Externalizing behavior										
*Erysipelatoclostridium*	− 648.0 (288.8)	0.027	0.051	− 649.8 (276.0)	0.020	0.039	1.00	1.00	< 0.1 (0.1)	15.2
*Holdemanella*	51.2 (15.2)	0.001	0.002	45.8 (14.7)	0.002	0.005	1.01	1.12	0.7 (1.6)	24.8
*Lachnospiraceae* ND3007 group	− 72.5 (26.4)	0.007	0.014	− 55.5 (28.5)	0.054	0.097	1.11	1.31	0.9 (1.0)	90.4
*Oscillospiraceae* NK4A214 group	81.6 (27.0)	0.003	0.006	67.2 (26.2)	0.012	0.023	1.02	1.21	0.6 (0.9)	84.8
*Phascolarctobacterium*	55.8 (21.2)	0.010	0.019	54.8 (20.6)	0.009	0.018	1.00	1.02	0.6 (1.2)	34.4
*Prevotella* 9	4.2 (2.0)	0.038	0.071	1.8 (2.0)	0.388	0.507	1.11	2.33	7.2 (12.9)	44.8
*Eubacterium* uncultured bacterium	57.1 (26.2)	0.031	0.059	52.3 (25.4)	0.042	0.077	1.01	1.09	1.0 (1.0)	79.2
Social anxiety										
*Collinsella*	− 4.6 (1.8)	0.012	0.024	− 3.7 (1.8)	0.045	0.083	1.07	1.24	0.7 (1.4)	52.0
*Erysipelatoclostridium*	80.5 (28.1)	0.005	0.010	73.8 (30.0)	0.016	0.030	1.02	1.09	< 0.1 (0.1)	15.2
*Faecalibacterium*	− 2.2 (0.5)	< 0.001	< 0.001	− 1.8 (0.5)	0.001	0.002	1.10	1.22	9.3 (5.0)	99.2
*Lachnospiraceae* ND3007 group	− 7.3 (2.8)	0.010	0.020	− 5.7 (3.0)	0.060	0.107	1.13	1.28	0.9 (1.0)	90.4
*Turicibacter*	13.4 (5.9)	0.026	0.049	8.6 (6.1)	0.160	0.251	1.03	1.56	0.2 (0.4)	32.0

Only microbial taxa, of which the original *p* < 0.05 in crude GLMs, are displayed here. In adjusted models, child gender, diet quality, and overnight sleep duration were included for internalizing behavior; overnight sleep duration and alcohol intake were included for externalizing behavior; and child gender, diet quality, overnight sleep duration, and paternal education levels were included for social anxiety. VIF < 3 indicates no multicollinearity in adjusted models

In crude GLMs, we found higher relative abundances of *Agathobacter*, *Lachnospira*, and *Turicibacter* in relation to more internalizing problems, while higher relative abundances of *Barnesiella* and *Faecalibacterium* were associated with less internalizing behavior (adjusted *p* < 0.05). However, none of them were significant after considering covariates.

With respect to externalizing behavior, in crude GLMs, we observed that higher relative abundances of *Holdemanella*, *Oscillospiraceae* NK4A214 group, *Phascolarctobacterium* were related to more externalizing behavior, while higher relative abundances of *Erysipelatoclostridium* and *Lachnospiraceae* ND3007 group were associated with fewer externalizing issues (adjusted *p* < 0.05, except for *Erysipelatoclostridium* with an adjusted *p* = 0.051). After accounting for covariates and multiple tests, the significance remained for *Holdemanella*, *Oscillospiraceae* NK4A214 group, and *Phascolarctobacterium*. Moreover, the estimate turned into significance for *Erysipelatoclostridium* in the adjusted model (adjusted *p* = 0.039 but with a low average relative abundance < 0.1%). Despite the insignificance, we noticed that the relation between *Prevotella* 9 and externalizing behavior changed strikingly after correcting for covariates (estimate from 4.2 to 1.8 with a fold change = 2.33).

Regarding social anxiety in crude GLMs, positive relations were observed for *Erysipelatoclostridium* and *Turicibacter*, while negative associations were found for *Collinsella*, *Faecalibacterium*, and *Lachnospiraceae* ND3007 (adjusted *p* < 0.05). After adjusting GLMs with covariates, differences remained significant for *Erysipelatoclostridium* and *Faecalibacterium*. Note that *Faecalibacterium* was highly prevalent across 14-year-old children (99.2%) with an average relative abundance at 9.3%.

## Discussion

In this study, we focused on a community sample of children in a longitudinal birth cohort (followed from 1 month to 14 years). Through the DMM method, we identified four distinct microbial clusters among these children in puberty, extending our knowledge on gut microbiota development and transition in this sensitive time window. By including microbial clusters determined in the first decade of life [8], we found that two *Prevotella* 9-enriched microbial clusters (i.e., Chilhood_2 and Puberty_2) were related to more externalizing behavior at age 14. Furthermore, Puberty_3, which was characterized by less *Faecalibacterium* compared to *Faecalibacterium*-enriched Puberty_1, was associated with more social anxiety at age 14. Additionally, higher relative abundances of *Faecalibacterium* were cross-sectionally linked to less social anxiety at age 14, supporting the Puberty_3 findings.

Our results indicated some similarities between microbial clusters in middle childhood and puberty [8]. Puberty_1 resembled Childhood_1 and similarly showed low phylogenetic diversity. Puberty_2 was predominated by *Prevotella* 9, and this was also a notable feature of Childhood_2. Furthermore, high phylogenetic diversity was observed in Puberty_3 and Puberty_4, seemingly in conformity with Childhood_3 and Childhood_4. Compared to the dynamic succession of microbial clusters in the first decade of life, the transition between pubertal clusters was steadier in this group of children. From the age of 12 to 14 years, most children within Puberty_1, Puberty_2, and Puberty_4 remained in the same clusters. Importantly, these three clusters were enriched in *Bacteroides*, *Prevotella* 9, and *Ruminococcus*, respectively, and this fits well with the three enterotypes reported in 2011, which seemed independent of age across different populations [50]. Therefore, it is possible that Puberty_1, Puberty_2, and Puberty_4 represent a more mature stadium of the gut microbiota. Conversely, Puberty_3 might correspond to a less mature phase, as its transition from age 12 was relatively divergent (i.e., the transition was almost equally towards Puberty_1, Puberty_2, and Puberty_3, indicating the presence of a more unstable cluster without a dominant transitional pattern).

Despite the weak differences between the genders in pubertal microbial clusters (i.e., the differences did not survive FDR corrections), some of these differences appear worth noting. For example, Puberty_2 and Puberty_4 tended to have more boys, while Puberty_3 tended to have more girls. Puberty_3 was enriched in *Bifidobacterium* with β-glucuronidase activity, able to deconjugate inactive bound estrogen into active non-bound estrogen [51]. Deconjugated estrogen can be reabsorbed by the gut and circulate in the bloodstream. After being conjugated by the liver, a portion of inactive estrogen reaches the gut and in turn may likely affect microbiota composition [52]. Estrogen, together with androgen, triggers the natural process of sexual maturation in puberty [53]. It has been suggested that gut microbiota composition may differ between disparate pubertal stages in a gender-dimorphic pattern [54, 55]. However, such discrepancy was not observed in our study, which considered pubertal status alone but not its interaction with child gender. Another unexpected finding was that general diet did not appear to explain the different pubertal clusters, while alcohol consumption did. At age 14, *Prevotella* 9-predominant Puberty_2 showed a higher ratio of consuming alcohol. This was in line with a recent finding that increased alcohol consumption, even moderate, was related to higher relative abundances of *Prevotella* 9 in adult populations [56]. Given the fact that sample size shrank after stratifying 14-year-old children based on microbial clusters and alcohol intake, our findings must be validated with another larger group of matched children.

Regarding microbial relations to behavioral measures, children within *Prevotella* 9-predominant Childhood_2 and Puberty_2 clusters exhibited more externalizing behavior at the age of 14 years. Although a positive cross-sectional relation was not observed between *Prevotella* 9 and externalizing behavior at age 14 after accounting for alcohol consumption and overnight sleep duration, such a trend conformed to our earlier findings in middle childhood (i.e., *Prevotella* 9 from ages 6 to 10 was positively related to child- and mother-reported externalizing behavior at age 10) [8]. In line with this, children with ADHD (attention deficit hyperactivity disorder), who are often characterized by impulsive and hyperactive externalizing symptoms, showed an overgrowth of *Prevotella* species including *P. amnii*, *P. buccae*, and *P. copri*, in comparison with typically developing children [57]. In particular, higher relative abundances of *P. buccae* were related to more impulsivity and hyperactivity problems, despite another study reporting less *Prevotella* in children with ADHD [58]. Furthermore, many ASD (autism spectrum disorder) cases show reductions in *Prevotella*, as concluded in a recent systematic review [59], while youth with early-life adversity (ELA) display higher relative abundances of *Prevotella* [60]. Before drawing any firm conclusions, we have to be aware of the wide species- and strain-level variability in *Prevotella*, which to a large extent may obscure the consistency between studies [61]. Moreover, potential influences of covariates (e.g., age, gender, diet, and lifestyle) and different etiologies behind mental problems should be considered carefully when comparing results.

More social anxiety was observed in microbial cluster Puberty_3, mainly at the age of 14 years. However, the most enriched taxa in this cluster (i.e., *Bifidobacterium*, *Akkermansia*, *Subdoligranulum*, *Christensenellaceae* R-7 group, and *Dialister*) were not cross-sectionally related to social anxiety at age 14. Nevertheless, higher *Bifidobacterium* has been frequently reported in MDD (major depressive disorder) [62], and lower *Subdoligranulum* and *Dialister* were found in GAD [24, 25], compared to healthy controls. When looking into other taxa, we found that lower *Faecalibacterium*, which was less enriched in Puberty_3 and highly prevalent at age 14, was associated with more social anxiety difficulties, in line with the finding of Puberty_3. Similarly, decreased *Faecalibacterium* has been observed in GAD patients [24], and related to increased duration and intensity of social exclusion experiences in young adults [63]. Furthermore, recent meta-analytic research described reduced *Faecalibacterium* in multiple mental disorders [62, 64], such as MDD, bipolar disorder, and ASD, despite a conflicting ASD result reported by another meta-analytic study [65]. As a gut commensal bacterium, *Faecalibacterium* is present in more than 90% of individuals in adult populations [66]. Its most studied and abundant species, *Faecalibacterium prausnitzii,* can produce anti-inflammatory molecules represented by butyrate [67]. Apart from regulation of inflammation, butyrate may suppress food intake and mediate cognition by influencing the concentrations of gut hormones [68]. Taken together, these findings suggest that *Faecalibacterium* may constitute a potentially important microbial marker for mental health.

A strength of our preregistered study is the use of a unique longitudinal community cohort followed from birth until age 14 years. This allows tracking gut microbiota development throughout infancy and childhood, assessing its predictive value for relevant behavioral measures in puberty. Importantly, we simplified the complex interplay between the gut microbiota and behavior by condensing the taxonomic data into identifiable microbial clusters. Furthermore, this study accounted for multiple potential covariates of behavioral measures when exploring their relations to the gut microbiota, decreasing the correlational bias to some extent. However, some limitations and perspectives should also be mentioned. First, the study was restricted by not considering the interaction of child gender with pubertal stages, mainly due to insufficient statistical power to further stratify groups. Second, although a collection of covariates was included, the gut microbiota and host behavior can still be affected by many unobserved or even unknown variables. Especially for an observational study, it is hence necessary to further validate the findings in another longitudinal community cohort or in carefully designed animal experiments. Third, once the conformity of correlational findings is validated, more attention can be given to the interpretation of functional potential of the gut microbiota, via gut-brain modules based on shotgun metagenomic sequencing or gut-metabolic profiles based on metabolomics [69]. Fourth, it remains a statistical challenge to explore relations between repeatedly measured microbiota data and a continuous numeric outcome variable. Currently, statistically sophisticated approaches to identify differentially abundant taxa over time were mainly created for group comparisons, such as SplinectomeR and zero-inflated beta regression methods [70]. Future research should aim to profile microbial trajectories across time and identify distinct ones [71], that can then be linked to host outcome phenotypes, or preferably, to host phenotypical development. Despite recent attempts at describing gut microbiota development, the step of associating variations in trajectories to host behavioral phenotypes is yet to be taken [4]. A final limitation of our study lies in the fact that 16S rRNA gene amplicon sequences are unable to provide results at the microbial species level.

Summarizing, in the current study, we identified four distinct microbial clusters in puberty, three of which were compositionally similar to enterotypes previously described at population level across different ages [50] and transitioned stably from age 12 to 14. Child gender may be a factor driving the formation of microbial clusters in puberty, although we did not find much evidence supporting this idea. The *Prevotella* 9-predominated clusters, including Childhood_2 and Puberty_2, were related to more externalizing behavior at age 14, while the *Faecalibacterium*-depleted Puberty_3 cluster was associated with more social anxiety at the same age. The cross-sectional negative relation between *Faecalibacterium* and social anxiety in 14-year-old children further supported this finding. Causal associations were not determined in this observational longitudinal study. Mechanistic research on a single taxon or an interactive group of taxa is needed to make it possible to describe causal relations between the gut microbiota and child pubertal mental health.

### Supplementary Information

Below is the link to the electronic supplementary material.

Supplementary file1 (PDF 36 KB)

Supplementary file2 (XLSX 43 KB)

## Data Availability

As the findings in this study are supported by datasets from an ongoing longitudinal cohort, these datasets currently cannot be made publicly available but are available upon request from C.deW. (Carolina.deWeerth@radboudumc.nl).

## Code availability

R codes can be accessed via this link https://doi.org/10.5281/zenodo.7529267
