# Supplementary material for: Development of the gut microbiota in the first 14 years of life and its relations to internalizing and externalizing difficulties and social anxiety during puberty
Source: Eur Child Adolesc Psychiatry. 2023 Apr 18;33(3):847–60. doi: 10.1007/s00787-023-02205-9 (PMC10894087; doi:10.1007/s00787-023-02205-9)
Supplement: Supplementary file 1 — Supplementary file1 (PDF 36 KB) [file 787_2023_2205_MOESM1_ESM.pdf]

Development of the gut microbiota in the first 14 years of life and its relations to internalizing and externalizing difficulties and social anxiety during puberty

European Child & Adolescent Psychiatry

Yangwenshan Ou[a,b], Clara Belzer[a], Hauke Smidt[a] & Carolina de Weerth[b]
[a]Laboratory of Microbiology, Wageningen University & Research, P.O. Box 8033, 6700 EH Wageningen, The Netherlands
[b]Radboud University Medical Center, Donders Institute for Brain, Cognition and Behaviour, Department of Cognitive Neuroscience, P.O. Box 9010, 6500 GL Nijmegen, The Netherlands
The corresponding author: Yangwenshan Ou (yangwenshan.ou@wur.nl)

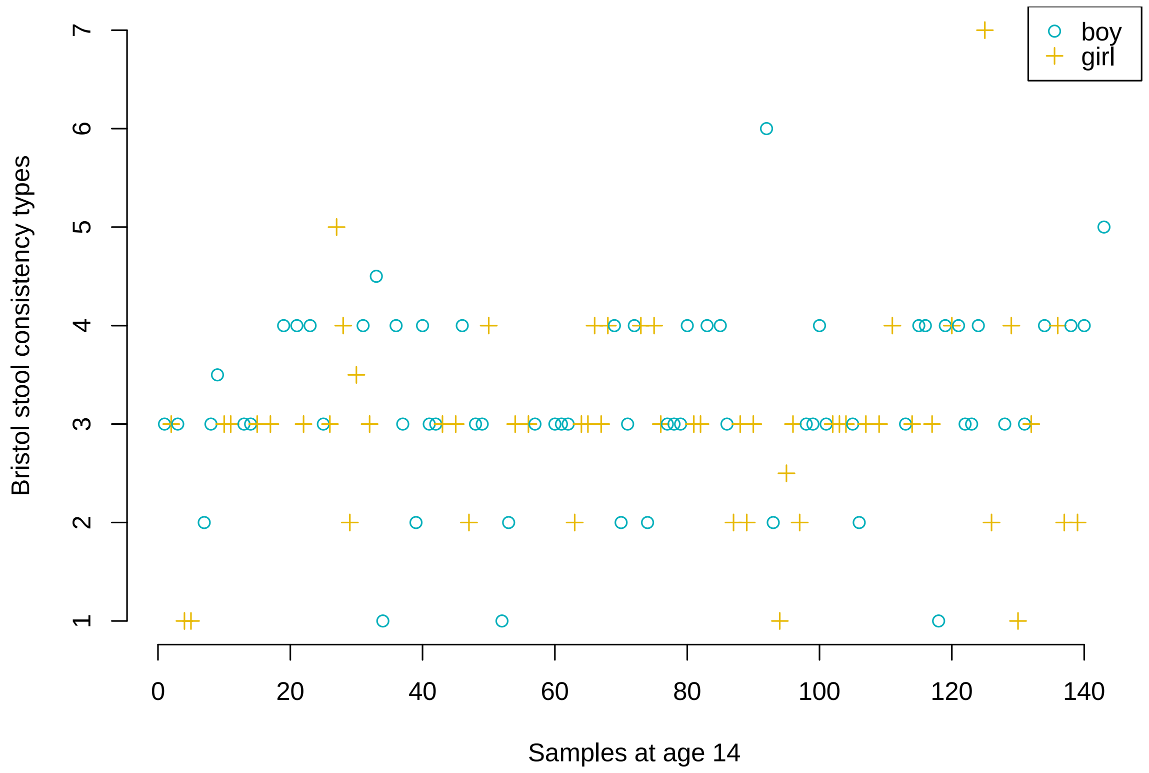

Figure S1. The distribution of Bristol stool consistency types of the samples at age 14. Among N = 125 participants providing stool samples at age 14, N = 7 (boy: girl = 3: 4) reported type 1 stools (separate hard lumps like nuts) during collection, N = 16 (boy: girl = 7: 9) reported type 2 stools (sausage shaped but lumpy), N = 1 girl reported a stool between types 2 and 3 (like a sausage or snake but with cracks on its surface), N = 58 (boy: girl = 29: 29) reported type 3 stools, N = 2 (boy: girl = 1: 1) reported stools between types 3 and 4 (like a sausage or snake, smooth and soft), N = 31 (boy: girl = 21: 10) reported type 4 stools, N = 1 boy reported a stool between types 4 and 5 (soft blobs with clear cut edges), N = 2 (boy: girl = 1: 1) reported type 5 stools, N = 1 boy reported a type 6 stool (fluffy pieces with ragged edges, a mushy stool), and N = 1 girl reported a type 7 stool (watery, no solid pieces).

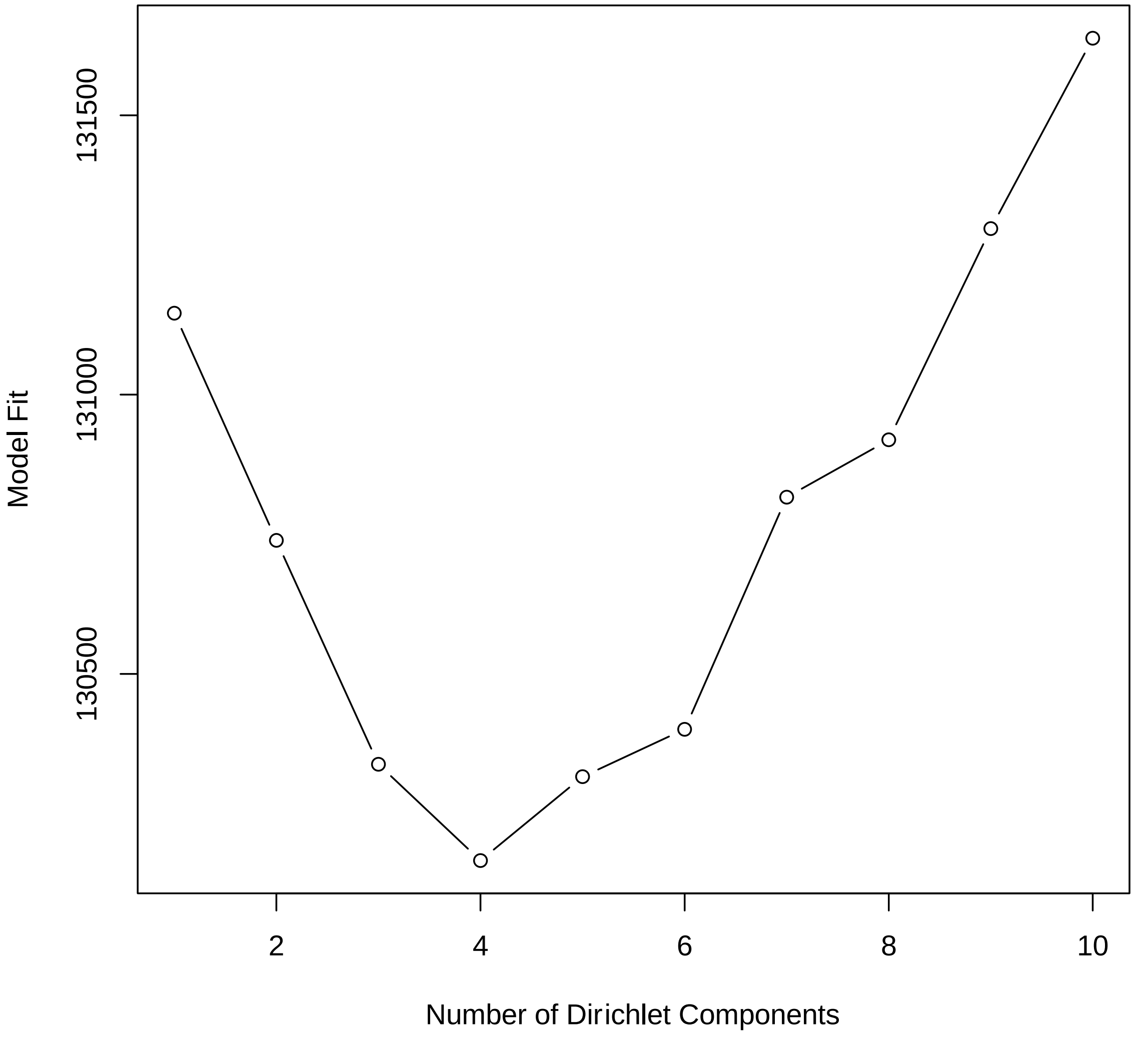

Figure S2. The number of Dirichlet components. Dirichlet Multinomial Mixtures models were conducted N = 10 times. Eighty percent (8/10) of the models showed consistent bacterial clusters of which the optimal number was four, as indicated by the lowest Laplace value.
